# “Worn-out but happy”: Postpartum Women's Mental Health and Well-Being During COVID-19 Restrictions in Australia

**DOI:** 10.3389/fgwh.2021.793602

**Published:** 2022-01-07

**Authors:** Hannah E. Christie, Kassia Beetham, Elizabeth Stratton, Monique E. Francois

**Affiliations:** ^1^School of Medicine, University of Wollongong, Wollongong, NSW, Australia; ^2^Illawarra Health and Medical Research Institute, University of Wollongong, Wollongong, NSW, Australia; ^3^School of Behavioural and Health Sciences, Australian Catholic University, Banyo, QLD, Australia; ^4^Faculty of Medicine and Health, Central Clinical School, University of Sydney, Sydney, NSW, Australia

**Keywords:** physical activity, post-pregnancy, pandemic, maternal, mood, exercise, depression, isolation

## Abstract

**Background:** From late 2019, COVID-19 disease has infiltrated the global population causing widespread challenges to public health. One cohort that has received less attention, but who may be more vulnerable to the mental and physical health related impacts of COVID-19 restrictions are postpartum mothers. The aim of this study was to explore the mental health, well-being, and health behaviours of mothers up to 12 months postpartum whilst living in Australia under COVID-19 level 3 and 4 restrictions.

**Methods:** 351 women in their first year postpartum residing in Australia whilst under level 3/4 social distancing restrictions (during April 13 and June 11, 2020) were recruited to participate in an online questionnaire. The survey measured symptoms of depression, anxiety, and stress (DASS), wellness (SF-36), physical activity (Godin-Shephard score), perceived value of health outcomes, diet, and sleep. Descriptive statistics and linear regressions were performed.

**Results:** Data was analysed for 139 eligible women. Of these women, 74% scored “normal” for depression, 84% for anxiety, and 72% for stress. Over half (58%) of women reported being worn out all, most, or a good bit of the time and 77% reported being a happy person all, most, or a good bit of the time. Analysis of the perceived values of health outcome revealed women had high value for “*getting out of the house,” “achieving a better overall mood,”* and “*to feel better physically*.” Women were considered physically active according to the Godin Leisure score, however only 41% of women met the current Australian national physical activity guidelines of 150 min.week^−1^.

**Conclusions:** Overall the majority of postpartum mums that were surveyed, have normal mental health symptoms, and despite being worn out most are happy at least a good bit of the time. This study highlights the importance of health values in maintaining leisure physical activity and mental health. In addition it appears women may benefit from virtual group exercise and community programs to encourage being physically active and socialising with friends simultaneously.

## Introduction

From late 2019, novel coronavirus disease (COVID-19) has infiltrated the global population causing widespread challenges to public health. The economic and social disruption has caused unprecedented stress and anxiety for many families. To minimise the spread of COVID-19 governments across the world, including Australia, have enforced various restrictions such as lockdown stay-at-home orders and the closure of many businesses, schools, and public spaces. Furthermore, restrictions in Australia included strict international and state border closures and limits on house and family visitation (1). The two highest levels of COVID-19 restrictions in Australia are the Level 3 and 4. These restrictions closed all services considered non-essential such as gym and fitness centres, dining, arts and entertainment, retail, and online home-schooling for most students. Reasons to leave the house were minimised to exercise (often time-restricted), essential shopping, and essential medical appointments. There has been much speculation on the impact of COVID-19 on mental health in the general population, with predictions of a 25% increase in suicides (2). One cohort that has received less attention, but who may be more vulnerable to the mental and physical health related impacts of disasters, such as, COVID-19 restrictions are postpartum mothers (3).

Notwithstanding the COVID-19 pandemic, the postpartum period poses many physical and emotional challenges and triumphs. In a cohort of ~10,000 Australian-born and migrant postpartum women, ~95% reported one or more emotional health issues within 6 months after birth; ~70% experienced extreme exhaustion (4), and 43% and 25% experienced heightened symptoms of depression and anxiety (5). These findings are of particular importance as links have been established between increased mental health symptoms and the impact on mother-infant bond as well as the psychological development of the infant (6). Achieving physical activity and diet guidelines are important during the postpartum period to improve cardiovascular fitness (7), facilitate weight loss (8), increase positive mood, decrease anxiety and depression, and promote greater alertness following exercise (9). Hence, it is important to explore the mental health and health behaviours of women during this pandemic in order to develop and strengthen support systems for postpartum mothers (10).

Recent studies across the world have reported mixed findings on the mental health and well-being of perinatal women during COVID-19 (11–18). Six studies in postpartum mothers across the world, North-eastern Italy, Belgium, China, and America have found increased depressive and anxiety symptoms (11, 12, 14–17), whilst another study in postpartum women in Japan reported normal levels of mental health and well-being (13). In addition, emerging evidence suggests the amount of physical activity women across the world partake in is also an important factor when considering maternal mental health during the pandemic (11). It is well-known that higher levels of physical activity can prevent (19) and reduce (20) mental health symptoms in the postpartum mother (21). However, due to COVID-19 restrictions there may be fewer opportunities for physical activity, potentially contributing to poorer mental health. One survey reported 64% of pregnant and postpartum women across the world decreased their physical activity levels during the COVID-19 pandemic, those who met the physical activity recommendations had lower levels of depression symptoms and anxiety compared to women who did not (11). Similarly, it is important to explore the impact of diet and nutrition. For example, recent research reports 54% of Australians were bothered by overeating during the pandemic (22). In addition, 43% of Danes have reported eating more, 42% have been snacking more, 48% are exercising less, and 30% have gained weight (23). To our knowledge, no studies have explored the impacts of COVID-19 restrictions on mental health and physical activity in postpartum women in Australia.

The aim of this study was to provide data on the mental health, well-being, and health behaviours of mothers up to 12 months postpartum whilst living in Australia under COVID-19 level 3 and 4 restrictions. It was hypothesised maternal mental health symptoms (depression, anxiety, and stress), and well-being would be lower than previously reported national averages. Physical activity levels were also expected to be low. The possible influence of important predictors of mental health such as physical activity, values and nutrition were also included as exploratory outcomes.

## Materials and Methods

### Sample and Procedure

Women >18 years, within their first year postpartum, and currently under Australian Government enforced Level 3 or 4 restrictions (24) were invited to complete an online questionnaire between April 13 and June 11 (all Australian states) and additionally, from September 10 to 22 (Victoria only) 2020. Women were recruited via advertisement on social media (Twitter, Facebook, and Instagram). This study was approved by the joint University of Wollongong and the Illawarra Shoalhaven Local Health District (ISLHD) Ethics Committee (HREC: 2019/ETH13571). Informed consent was provided by all women.

#### Instruments

The questionnaire and consent were completed by women via the online platform CoreXM (Qualtrics, Sydney, Australia). Demographics including such as age, months since delivery, education, and postcode were collected, followed by questions on well-being, mental health, and health related behaviours.

##### Mental Health

Mental health symptoms were collected via the *Depression, Anxiety, and Stress Scale*−*21 items* [*DASS-21* (25)]; a set of three self-report scales assessing seven-items across anxiety, depression, and stress symptoms. Mental health is analysed and reported categorically using the classifications provided in the DASS: normal, mild, moderate, severe, and extremely severe. The short version has good reliability and validity (Cronbach's alpha 0.94, 0.87, and 0.91 for the depression, anxiety, and stress subscales, respectively), along with good construct validity.

##### Well-Being

Well-being was assessed using the *36-item Short Form Health Survey questionnaire (SF-36)*. The SF-36 is a validated instrument for evaluating Health-Related Quality of Life. The SF-36 measures eight domains of health status: physical functioning (10 items), physical role limitations (four items), bodily pain (two items), general health perceptions (five items), energy/vitality (five items), social functioning (two items), emotional role limitations (three items), and mental health (five items). This version has good reliability and validity (Cronbach's alpha for physical functioning: 0.93; social functioning: 0.73; physical role limitations: 0.96; emotional role limitations: 0.96; bodily pain: 0.85; mental health: 0.95; energy/vitality: 0.96; and general health perceptions: 0.95) (26). The scores were transformed to score from zero (worst possible health) to 100 (best possible health) using RAND (Research and Development) Corporation's scoring guidelines (27).

##### Perceived Value of Outcomes

Perceived value of outcomes for general health was assessed using a 12-item outcome expectations measure designed to be relevant to the study population. Participants were asked to rate items by “*How much value do you place on attaining each of the following”* followed by items such as “*get out of the house,” “weight control,” and “lower risk of type 2 diabetes” (****Table 5****)*. Each item was scored on a 9-point Likert scale ranging from 1 (no value) to 9 (the highest of value) (28). The questionnaire has good validity and reliability (Cronbach's alpha for this study: 0.82).

##### Sleep

Sleep was assessed using a 4-item questionnaire evaluating the length of sleep, the length of uninterrupted sleep, and awake hours during night (all measured in hours) as well as the number of interruptions. Interruptions were assessed on a categorical scale of 0–1, 2–3, 4–5, 6–7, and 7 or more-times sleep was interrupted per night. The questionnaire has good validity and reliability (Cronbach's alpha for this study: 0.73).

##### Physical Activity

Physical activity was assessed using the validated *Godin-Shephard Leisure-Time Physical Activity Questionnaire* [Godin Score, (29); kappa index 0.74 (30)]. The questionnaire asked participants to report how often they completed 15 min bouts of strenuous, moderate, and light/mild exercise over a typical 7-day period before, and during their pregnancy, as well-currently (postpartum). These values were then placed into the following equation to provide their leisure score separately during each time period:


$$\begin{array}{l} Weekly\;leisure\;activity\;score=\left(9\;\times \;Strenuous\right)\\ \;+\left(5\;\times \;Moderate\right)+\left(3\;\times \;Light\right) \end{array}$$

Godin scores are categorised into active (score: ≥ 24), moderately active (score: 14–23), or insufficiently active (score: ≤ 13). Physical activity minutes per week were then calculated from the reported Godin scores.

##### Dietary Intake

*Dietary intake* of the core food groups [including vegetables (fresh), vegetables (frozen), fruit (fresh), fruit (frozen), grains, legumes, meat, dairy, and snacks] were assessed using a questionnaire developed in line with Australian Dietary Guidelines (31). Each item was scored on a 4-point scale including 1 (daily), 2 (3–4 days.week^−1^), 3 (weekly), and 4 (rarely). Food groups were separately analysed. Dietary intake was compared with the Australian Dietary Guidelines (31).

##### Data Analysis and Statistics

SPSS Version 26.0 (Armonk, NY, USA) was used for quantitative analysis. Univariate data for baseline demographics, mental health, physical activity, sleep, dietary intake, and wellness outcomes are shown as a proportion (percentage) or mean (SD). Correlations of all variables were carried prior to regression analysis (Supplementary Tables 1–7). Multiple linear regression analysis was used to explore the relationship between mental health score (dependent variable) and predictor variables-education, postpartum BMI, physical activity, and food intake (fresh vegetables, fresh fruits). Multiple linear regression was also used to establish the relationship between postpartum physical activity (dependent variable) and the above predictors with the addition of pregnancy physical activity levels. Linear regression was also used to determine the relationship between postpartum physical activity (dependent variable) and values for general health. Variables used in the regression analysis (education, BMI, maternal age, fruit and vegetables and physical activity) were based on known relationships of between variables and mental health/physical activity. Regression tables are presented with standardised coefficients β, t-statistic (t), significance (p), and 95% confidence intervals for β (upper and lower bounds). Statistical significance was noted as *p* ≤ 0.05. Participant data was only used for those participants that completed each section (ie mental health, well-being etc) in full, incomplete sections were excluded from analysis. *Post-hoc* power analysis for mental health (depression, anxiety, and stress) revealed a sample size of 118 was required.

## Results

A total of 351 participants expressed interest in the study. Of the 351, 212 met inclusion criteria but did not complete the survey for unknown reasons. Data for 139 eligible participants were available for most analyses. However, where fewer women completed a survey section (i.e., DASS *n* = 114) the sample size is reported. Women had a mean age of 32.5 ± 4.2 years, were 6.2 ± 3.6 months post-delivery, and had a mean postpartum BMI of 25.8 ± 4.2 kg.m^−2^ (Table 1). Of these women, 40% were primiparous, 44% had two children, 3% had 3 children, and 2% had 4 children. Further, 85% were married, and 80.3% had at least a bachelor degree.

**Table 1:** Physical activity, body mass index (BMI), sleep, dietary intake, and perceived value of achieving outcomes of women reported pre-, during, and post-pregnancy (during COVID-19 social distancing restrictions).

	**Pre-pregnancy** **mean ± SD**		**Pregnancy** **mean ± SD**		**Post-partum** **mean ± SD**
Physical activity (min/wk)	149.1 ± 58.9		124.7 ± 52.9		133.5 ± 58.8
Godin score	52.6 ± 22.9		40.3 ± 18.5		43.6 ± 20.4
Strenuous	2.7 ± 1.9		1.6 ± 1.3		1.9 ± 1.5
Moderate	3.4 ± 2.0		2.8 ± 2.0		3.0 ± 2.3
Light	3.9 ± 2.2		3.9 ± 2.2		4.1 ± 2.4
BMI (kg.m^−2^)	24.4 ± 4.1		13.2 ± 6.0* *pregnancy weight gain (kg)		25.8 ± 4.2
**Postpartum sleep**	**0–1%**	**2–3%**	**4–5%**	**6–7%**	**7+%**
Hours of sleep total	0	0.8	24.4	53.5	21.3
Uninterrupted sleep (hours)	7.9	49.6	28.3	9.4	4.7
How many times sleep interrupted? (*n*)	16.5	45.7	22.0	4.7	11.0
Awake time during night (hours)	15.7	61.4	20.5	1.6	0.8
**Food**	**Daily %**	**3–4 times weekly %**		**Weekly %**	**Rarely %**
Vegetables (Fresh)	66.7	28.1		3.7	1.5
Vegetables (Frozen, dried, or canned)	14.4	23.2		30.4	32.0
Fruit (Fresh)	51.2	24.4		18.1	6.3
Fruit (Frozen, dried, or canned)	9.6	6.4		24.0	60
Grains	70.6	21.4		4.8	3.2
Meat	71.7	25.2		1.6	1.6
Legumes	19.0	24.6		37.3	19.0
Dairy	71.7	14.2		7.9	6.3
Snacks	32.3	26.8		33.1	7.9
	**Mean ± SD**				
Get myself out of the house	7.8 ± 1.6				
Feel better physically	7.7 ± 1.5				
Better overall mood	7.5 ± 1.7				
Sense of accomplishment	7.3 ± 1.7				
Reduce stress	7.1 ± 2.0				
Socialise with friends	7.1 ± 1.8				
Have more energy	7.0 ± 1.9				
Increase fitness	6.8 ± 2.0				
Weight control	6.4 ± 2.2				
Lower risk of type 2 diabetes	4.8 ± 2.8				
Meet new people	4.4 ± 2.0				
Praise from friends and family	4.1 ± 2.5				

### Mental Health and Well-Being

A majority of women reported normal scores in depression, anxiety, and stress symptoms during Australian level 3 and 4 social distancing restrictions. Women had a mean depression score of 3.16 ± 3.08. Of these, 74% (*n* = 84/114) scored as normal, 11% (*n* = 12/114) mild, 12% (*n* = 14/114) moderate, and 4% (*n* = 4/114) severe depression. No participants scored extremely severe range.

Women in the study had a mean anxiety score of 2.09 ± 2.80. Of these, 84% (*n* = 94/112) scored normal, 7% (*n* = 8/112) mild, 6% (*n* = 6/112) moderate, 4% (*n* = 4/112) severe, and 4% (*n* = 4/112) extremely severe anxiety.

Women in the study had a mean stress score of 6.14 ± 3.95. Of these, 72% (*n* = 82/114) scored normal, 11% (*n* = 12/114) mild, 9% (*n* = 10/114) moderate, 8% (*n* = 9/114) severe, and 1% (*n* = 1/114) extremely severe stress.

SF-36 scores, separated into the eight core wellness concepts (physical functioning, physical role, bodily pain, general health, vitality, social functioning, emotional role, and mental health), are provided in Table 2. Compared to Australian norms, our population had significantly higher levels of physical functioning (94.8 ± 6.9 vs. 89.3 ± 23.4 u.a; *p* = 0.010) and lower levels of general health (65.3 ± 18.6 vs 75.9 ± 28.0 u.a.; *p* < 0.001), vitality (45.4 ± 19.0 vs. 62.3 ± 28.0 u.a.; *p* < 0.001), and emotional role (60.9 ± 38.9 vs. 83.7 ± 46.7 u.a.; *p* < 0.001). Within these concepts, 58.1% of women reported being worn out all, most, or a good bit of the time and 76.9% of women reported being a happy person all, most, or a good bit of the time.

**Table 2:** Postpartum well-being of women as measured through an SF-36 (*n* = 121), compared with Australian female norms aged 25–34 years (*n* = 2,182).

**SF-36**	**Mean ± SD (a.u)**	**Australian norms, females 25–34 years (32)**	***P*-value**
Physical functioning	94.8 ± 6.9	89.3 ± 23.4	0.010^*^
Physical role limitations	78.6 ± 28.2	83.5 ± 46.7	0.253
Bodily pain	74.2 ± 22.0	79.8 ± 32.7	0.063
General health	65.3 ± 18.6	75.9 ± 28.0	<0.001^*^
Vitality	45.4 ± 19.0	62.3 ± 28.0	<0.001^*^
Social functioning	78.5 ± 23.5	84.0 ± 32.7	0.068
Emotional role limitations	60.9 ± 38.9	83.7 ± 46.7	<0.001^*^
Mental health	70.0 ± 16.6	74.2 ± 23.4	0.052

*Missing values due to incomplete datasets*.

* *Significance < 0.05*.

### Sleep

Half of the women (53.5%) reported getting 6–7 h of sleep per night total. Many women (49.6%) reported getting 2–3 h of uninterrupted sleep each night and 45.7% reporting being interrupted 2–3 times a night. 61.4% of women reported spending 2–3 h awake during the night. Sleep frequencies are provided in Table 1.

### Perceived Value of Outcomes

The average perceived value (1–low value to 9–highest value) placed on achieving outcomes of general health are shown in Table 1. In regard to value toward various health components, postpartum women reported high value for “*getting out of the house,”* achieving a “*better overall mood,”* and “*to feel better physically*.” Lowest value was reported for “*receiving praise from family and friends,” “meeting new people,” and “lowering the risk of type 2 diabetes.”*

### Physical Activity

On average, women in the present study were classified as being physically active pre-, during and postpartum according to the Godin classification (average min.wk^−1^ Table 1). Pre-pregnancy, women had a Godin score of 53 ± 23 a.u. Of these, 0% were classed as sedentary or physically inactive, 7% (*n* = 9/125) moderately active, and 93% (*n* = 116/125) physically active according to the Godin score.

During pregnancy, women had a mean Godin score of 40 ± 19 a.u. 0% (*n* = 0/121) were classed as sedentary or physically inactive, 18% (*n* = 22/121) were considered moderately active, and 82% (*n* = 99/121) were considered physically active according to the Godin score.

Postpartum, during Australian level 3 and 4 COVID-19 restrictions, women had a Godin score of 44 ± 20 a.u. Of these, 0% (*n* = 0/121) were classed as sedentary or physically inactive, 16% (*n* = 19/121) moderately active, and 84% (*n* = 102/121) physically active according to the Godin score. Despite the positive scores derived from the Godin scores, only 41% of women met the current physical activity guidelines of 150 min.week^−1^ according to Australian national guidelines (33). Godin scores for vigorous, moderate, and light physical activity is presented in Table 1.

### Nutrition

Women in the study reported eating fresh vegetables (66.7%), fresh fruit (51.2%), grains (70.6%), meat (71.6%), and dairy (71.7%) daily. Frozen fruit and vegetables were reported as being eaten rarely (60.0 and 32.0%, respectively). Legumes were most commonly (37.3%) reported as being eaten weekly, whilst snacks were reported as being eaten both daily and weekly (32.3 and 33.1%, respectively). Based on this survey, the proportion of women in this study that do not meet healthy eating guidelines is 33.3% for vegetables, 48.8% for fruits, 29.4% for grains, 28.8% for meats, 81% for legumes, and 28.3% for dairy. The proportion of women who answered daily, 3–4x per week, weekly or rarely to each food group is shown in Table 1.

### Relationship Between Physical Activity and Maternal Mental Health, Values, and Well-Being

Correlation analysis was performed on all variables, and significant outcome variables were entered into regression analysis (Supplementary Tables 1–7). Several regression analyses were conducted with depression, anxiety, and stress scores with education, postpartum BMI, vegetable intake, fruit intake, pre-pregnancy physical activity, and postpartum physical activity as potential predictors. Fruit intake (*p* = 0.039) was a significant predictor for depression symptoms and accounted for 12.6% of depression score variance (Table 3). Fruit intake (*p* < 0.001) was a significant predictor for anxiety symptoms and accounted for 26.8% of anxiety score variance (Table 3). Fruit intake (*p* = 0.007) was a significant predictor for stress symptoms and accounted for 17.1% of stress score variance (Table 3). Physical activity carried out pre-pregnancy (*p* = 0.050) and during pregnancy (*p* = 0.001) were both significant predictors for postpartum physical activity and together accounted for 40.4% of postpartum physical activity time (Table 4). Value in feeling better physically (*p* = 0.006), getting out of the house (*p* = 0.034) and socialising with friends (*p* = 0.033) were all significant predictors for postpartum physical activity and together accounted for 16.9% of post-pregnancy physical activity time variance (Table 5).

**Table 3:** Linear regression analysis for mental health scores against education level, post-partum BMI, fresh vegetable and fruit intake, pre-pregnancy physical activity, postpartum physical activity.

	**Standardised coefficients beta**	***T***	**Sig**	**95% confidence interval lower bound**	**95% confidence interval upper bound**
**Depression**					
Constant		0.35	0.729	−3.52	−5.01
Education	0.01	0.05	0.957	−0.64	0.68
Postpartum BMI	−0.06	−0.60	0.548	−0.18	−0.09
Vegetables: fresh	0.21	1.92	0.058	−0.04	2.13
Fruit: fresh	0.23	2.10	0.039^*^	0.04	1.53
Pre-pregnancy PA min	0.01	0.08	0.936	−0.01	0.015
Postpartum PA min	0.10	0.83	0.411	−0.01	0.02
*F*_(6, 89)_ = 2.147; *p* = 0.056; *R^2^* = 0.126					
**Anxiety**					
Constant		0.55	0.586	−2.48	4.36
Education	−0.18	−1.89	0.063	−1.03	0.03
Postpartum BMI	−0.02	−0.21	0.835	−0.12	0.10
Vegetables: fresh	0.14	1.35	0.180	−0.28	1.46
Fruit: fresh	0.39	3.82	<0.001^*^	0.55	1.74
Pre-pregnancy PA min	0.06	0.54	0.590	−0.01	0.02
Postpartum PA min	−0.09	−0.84	0.403	−0.02	0.01
*F*_(6, 89)_ = 5.423; *p* < 0.001; *R^2^* = 0.268					
**Stress**					
Constant		1.03	0.307	−2.53	7.97
Education	0.04	0.39	0.695	−0.65	0.98
Postpartum BMI	−0.10	−0.96	0.340	−0.25	0.09
Vegetables: fresh	0.21	1.96	0.053	−0.02	2.66
Fruit: fresh	0.30	2.76	0.007^*^	0.36	2.19
Pre-pregnancy PA min	0.01	0.05	0.957	−0.02	0.02
Postpartum PA min	0.14	1.19	0.239	−0.01	0.03
*F*_(6, 89)_ = 3.055; *p* = 0.009; *R^2^* = 0.171					

* *p < 0.05*.

**Table 4:** Linear regression analysis for postpartum physical activity against education level, post-partum BMI, Fresh vegetable and fruit intake, pre-pregnancy physical activity, pregnancy physical activity.

	**Standardised coefficients beta**	***T***	**Sig**	**95% confidence interval lower bound**	**95% confidence interval upper bound**
**Postpartum physical activity (minutes)**					
Constant		1.59	0.116	−11.71	104.63
Education	0.00	0.01	0.992	−9.20	9.29
Postpartum BMI	−0.02	−0.19	0.847	−2.06	1.69
Vegetables: fresh	−0.08	−0.89	0.378	−22.69	8.69
Fruit: fresh	−0.01	−0.13	0.894	−11.61	10.15
Pre-pregnancy PA min	0.24	1.99	0.050^*^	0.00	0.49
Pregnancy PA min	0.40	3.27	0.001^*^	0.18	0.72
*F*_(6, 99)_ = 11.178; *p* < 0.001; *R^2^* = 0.404					

* *p < 0.05*.

**Table 5:** Linear regression analysis for postpartum physical activity against reported values for general health.

	**Standardised coefficients beta**	***T***	**Sig**	**95% confidence interval lower bound**	**95% confidence interval upper bound**
**Postpartum physical activity (minutes)**					
Constant		0.84	0.401	−43.88	108.77
Get out of the House	0.22	2.15	0.034^*^	0.61	15.25
Feel better physically	0.41	2.79	0.006^*^	4.50	26.85
Better overall mood	−0.12	−0.079	0.434	−16.57	7.17
Reduce stress	0.20	1.38	0.172	−2.66	14.71
Sense of accomplishment	−0.21	−1.66	0.100	−17.02	1.52
Gain more energy	−0.20	−1.40	0.164	−15.35	2.64
Lower the risk of type 2 diabetes	0.15	1.37	0.174	−1.34	7.27
Praise from family and friends	0.05	0.51	0.613	−3.40	5.74
Socialise with friends	−0.22	−2.16	0.033^*^	−13.79	−0.58
*F*_(9, 97)_ = 2.184; *p* = 0.029; *R^2^* = 0.169					

* *p < 0.05*.

## Discussion

This study is the first to explore the mental health, well-being, and physical activity levels of postpartum (<1 year) mothers living in Australia during COVID-19 level 3 and 4 restrictions. The present study found that despite feeling worn out, and being sleep-deprived, postpartum women during COVID-19 reported (i) normal symptoms of mental health (depression, anxiety, and stress) and well-being, and (ii) were classed as being physically active postpartum (that being during COVID restrictions) according to the Godin Leisure questionnaire. Of interest, the women held high value for achieving a “better overall mood” and “feeling better physically,” even though they reported having normal mental health symptoms and well-being. The value placed upon feeling better physically was positively correlated to the minutes of postpartum physical activity women achieved during COVID-19 restrictions. Fruit and vegetable intake were also positive predictors of better mental health. Taken together, our findings highlight the benefits of maintaining physical activity and healthy eating, and the importance of womens' value toward achieving a better mood and feeling better physically, during a global pandemic such as COVID-19; at a time when barriers to maintaining emotional and physical behaviours are high. This is in line with other COVID-19 research that has found physical activity participation correlates with lower depression and anxiety scores (11) and that perceived stress is related to how women perceive the rewards over challenges, during COVID-19 restrictions (34).

Women in the present study had higher proportions of normal mental health symptoms compared to a recent international survey also conducted during COVID-19 (comprising of pregnant and postpartum participants mostly from North America) (11). In contrast with that study, we did not find strong relationships between physical activity (whether pre-pregnancy, or postpartum) and mental health. Based on prior research in pregnant and postpartum mums internationally (11, 35, 36), it was anticipated that social distancing and isolation measures during COVID-19 restrictions would negatively impact the mental health of Australian postpartum mothers. Previous international research during COVID-19 has been mixed reporting depressive symptoms in the general population were worse (11, 35), better (37) or the same (13). Worsening mental health symptoms were reported by countries such as Southern Italy and North America, where the number of cases and fatalities were much higher than Australia and stricter levels of stay-at-home restrictions (i.e., only one household member able to shop for food) (11, 12). However, our study found Australian women's depression scores (DASS; 26% with values categorised not “normal”) were similar to a survey in North-Eastern Italy [Edinburgh Postnatal Depression Score (EPDS): 28.6% above 12], despite Italy having more cases of COVID-19 and having stricter isolation restrictions than Australia (12). Further investigation into the sociocultural differences is warranted as a potential explanation rates of depression in Italy despite worse COVID-19 conditions. Furthermore, relationships were found between improved mental health and daily fruit and vegetable intake though future research may begin to further investigate the intricacies of this relationship.

The health values' women hold is important as they drive health behaviours which may be compromised during the COVID-19 pandemic. During the COVID-19 pandemic postpartum women held the highest value for *getting out of the house, feeling better physically*, and *improving overall mood*. Correlations have previously been established between moderate physical activity and improved mood in healthy populations (38, 39). The value women in this study placed on feeling better physically was associated with more minutes per week of physical activity, which, based on the above relationship, will likely lead to improved mental health. Indeed, prior research has already shown that higher levels of physical activity are associated with better mental health during the COVID-19 pandemic (12). A small, but significant relationship was identified between having a high value toward socialising with friends and lower levels of physical activity during COVID-19 restrictions. This suggests women were not participating in physical activity whilst socialising with friends during COVID restrictions. Here, women were likely socialising over social media or video conferencing, which in turn may encourage sustained sedentary behaviours. Strategies to reach physical activity guidelines whilst socialising with others may be of importance to women during the (or a future) pandemic. For example, future research should explore whether women would benefit from virtual group exercise and community programs to encourage being physically active and socialising with friends simultaneously.

It is well-known that regular participation in physical activity can improve and maintain mental health and well-being (19–21), however, many women fail to maintain sufficient physical activity during and following pregnancy (40). Based on previous research (11, 36) and given the closure of gyms and fitness facilities in Australia, it was widely anticipated that physical activity would be low during COVID-19 social distancing measures. Despite women reporting being physically active on the Godin Leisure questionnaire, on average, only 41% of the postpartum women we sampled were meeting Australian physical activity guidelines of 150 min.week^−1^ during COVID-19 social distancing restrictions. This is lower than previously reported in Australia prior to the pandemic (2008–2010) which reported 63% of postpartum women in Australia meet the physical activity guidelines (40). Interestingly, one study reported 76% of pregnant women in the United States had no change in physical activity throughout lockdown protocols (41), though this may be due to the stringency of the lockdown enforcements at the time of data collection. Mothers in this study, whilst not meeting traditional structured exercise guidelines, may actually still be active and regularly move (a common occurrence when looking after small children), thereby meeting the physical activity leisure guidelines, which still likely have health enhancing benefits. The findings from this study provide important impetus for future research investigating the benefits of regular active movement objectively (i.e., measured by steps/day) verses structured physical activity for mental health and well-being.

### Strengths, Limitations, and Future Directions

This is the first study to explore health behaviours in mothers in Australia (<1 year postpartum) during COVID-19 social distancing restrictions. Whilst this was one of the first studies internationally to investigate the wellness, physical activity and dietary patterns of postpartum women during COVID-19, the diet questionnaire used has not been validated. Future research which collects food records may provide more specific and accurate data regarding nutrition in a pandemic. Our study did not account for a control (pre-pandemic group), however given this was not possible (without asking women to recall retrospectively) we have compared our findings to large-scale data, validated, Australian population norms. Future research may benefit in examining the health behaviours of women during and post the COVID-19 restrictions (42, 43). The exploratory regression relationships between outcomes require further research given our small sample size. In addition to differing quarantine levels and number of cases, discretions in mental health might also be due to the level of support, physical activity and health habits, values and outcome expectations, and the type and timing of postpartum data collection. Regardless, this study shows that even in times of additional barriers, such as with the COVID-19 lockdown and restrictions, women can still maintain physical activity, health, and well-being, if they place value on these parameters and therefore future research should be placed on increasing these values through strategies such as community programs.

## Conclusions

In general, postpartum mums in Australia had normal mental health symptoms during COVID-19 restrictions. This study provides evidence for the importance of health values in maintaining mental health and physical activity during times of additional barriers, such as the COVID-19 pandemic. Results provided suggest if postpartum women in the Australian population can be educated on the benefits of physical activity and well-being and thus find value in improving mood and feeling better physically, they may also be able to reach physical activity guidelines and overcome any barriers faced during the COVID-19 pandemic. Future research should explore the potential for a virtual community group exercise program to encourage women to combine socialising and physical activity for improved mental health. The provision of specialists in exercise physiology and nutrition may encourage women to place a value on attaining greater fitness, and subsequently improve physical and mental health.

## Data Availability Statement

The raw data supporting the conclusions of this article will be made available by the authors, without undue reservation.

## Ethics Statement

The studies involving human participants were reviewed and approved by University of Wollongong Human Ethics Research Committee. The patients/participants provided their written informed consent to participate in this study.

## Author Contributions

HC and MF designed the trial and wrote the original manuscript. HC carried out recruitment and data collection. HC, MF, ES, and KB carried out data analysis. All authors edited and approved the final manuscript.

## Funding

MF was supported by an Australian National Health and Medical Research Council (NHMRC) Investigator grant.

## Conflict of Interest

The authors declare that the research was conducted in the absence of any commercial or financial relationships that could be construed as a potential conflict of interest.

## Publisher's Note

All claims expressed in this article are solely those of the authors and do not necessarily represent those of their affiliated organizations, or those of the publisher, the editors and the reviewers. Any product that may be evaluated in this article, or claim that may be made by its manufacturer, is not guaranteed or endorsed by the publisher.

## Abbreviations

DASS: Depression, anxiety, stress scale
EPDS: Edinburgh postnatal depression scale
BMI: Body mass index
SF-36: 36-item short form survey.
