# Supplementary material for: “Worn-out but happy”: Postpartum Women's Mental Health and Well-Being During COVID-19 Restrictions in Australia
Source: Front Glob Womens Health. 2022 Jan 7;2:793602. doi: 10.3389/fgwh.2021.793602 (PMC8777212; doi:10.3389/fgwh.2021.793602)
Supplement: Supplementary file 1 [file Table_1.docx]

Supplementary Table 1. Correlation table for demographics and all variables. * Significance p = 0.05; ** significance p = 0.01.

|  | Age | Relationship Status | Occupation | Education | Parity | Child Age | Pre-pregnancy BMI | Gestational Weight Gain | Post-pregnancy BMI |
| --- | --- | --- | --- | --- | --- | --- | --- | --- | --- |
| Age | 1 |  |  |  |  |  |  |  |  |
| Relationship Status | -.42** | 1 |  |  |  |  |  |  |  |
| Occupation | .00 | .05 | 1 |  |  |  |  |  |  |
| Education | .28** | .26** | -.13 | 1 |  |  |  |  |  |
| Parity | .24** | .25** | -.13 | .16 | 1 |  |  |  |  |
| Child Age | .04 | -.03 | .33** | .00 | -.16 | 1 |  |  |  |
| Pre-pregnancy BMI | .24** | .18* | .13 | -.03 | -.57 | -.01 | 1 |  |  |
| Gestational Weight Gain | .15 | -.04 | .05 | -.04 | -.19* | .33** | .25** | 1 |  |
| Post-pregnancy BMI | -.25** | .17 | .08 | -.02 | .01 | -.01 | .92** | -.09 | 1 |
| Pre-pregnancy Physical Activity | -.07 | .15 | -.04 | .18* | .00 | -.14 | -.05 | -.06 | -.07 |
| Pregnancy Physical Activity | .02 | .01 | -.14 | .19* | .07 | -.12 | -.10 | -.11 | -.02 |
| Post-pregnancy Physical Activity | -.08 | .10 | -.00 | .10 | -.04 | -.02 | .10 | -.22* | -.06 |
| Vegetables (Fresh) | -.25** | .06 | .00 | -.09 | .02 | .00 | .14 | -.01 | -.21* |
| Vegetables (Frozen) | .02 | -.05 | -.20* | .05 | -.00 | .07 | -.17 | -.04 | -.19* |
| Fruit (Fresh) | -.16 | .06 | -.02 | -.05 | -.04 | -.11 | .20* | .03 | .27** |
| Fruit (Frozen) | -.00 | -.15 | -.21* | .16 | .13 | .03 | .05 | -.18 | .01 |
| Grains | .06 | .03 | .09 | -.17 | .01 | .09 | -.01 | .12 | -.06 |
| Meat | -.02 | -.09 | .01 | .10 | .07 | -.07 | .08 | .01 | .08 |
| Legumes | .21* | .14 | -.12 | -.08 | -.07 | -.02 | .09 | -.09 | .15 |
| Dairy | -.06 | -.01 | .04 | -.10 | -.14 | .16 | -.06 | .12 | .05 |
| Snacks | .01 | .11 | -.08 | -.16 | -.07 | .19* | -.08 | .16 | -.17 |
| Hours of Sleep (Total) | -.05 | -.14 | .10 | -.02 | -.09 | .06 | -.03 | .08 | -.07 |
| Hours of Sleep (Uninterrupted) | .03 | .13 | .27** | -.11 | -.14 | .22* | .14 | .12 | .08 |
| Number of Sleep Interruptions | -.09 | .10 | -.16 | -.08 | .07 | -.17 | -.01 | -.00 | .05 |
| Hours Awake at Night | -.91 | .18* | -.18* | -.06 | -.01 | -.29** | .06 | -.05 | .07 |
| Socialising with friends | .18 | -.05 | -.05 | .03 | -.04 | .04 | -.11 | .02 | -.08 |
| Getting Out of the House | .12 | -.03 | -.15 | .04 | -.15 | -.02 | .07 | -.15 | -.09 |
| Meet New People | .08 | .07 | -.19* | .03 | -.15 | -.12 | -.13 | -.09 | -.05 |
| Praise | -.37 | .04 | -.02 | -.09 | -.11 | -.05 | .07 | -.06 | .01 |
| Weight Control | .15 | -.09 | .07 | -.11 | .10 | -.05 | .10 | .03 | .08 |
| Increase Fitness | .24* | -.04 | .04 | .01 | .11 | .00 | .02 | .02 | -.09 |
| Reduce the risk of Type 2 Diabetes | -.01 | .03 | .09 | -.13 | .07 | -.05 | .20* | -.23 | .21* |
| More Energy | .18 | .08 | .41 | -.16 | .27** | .01 | .14 | -.03 | .11 |
| Feel Better Physically | .19* | -.01 | .01 | -.02 | .16 | -.03 | .18 | -.07 | .12 |
| Reduce Stress | .17 | -.06 | -.06 | -.15 | .16 | .08 | -.04 | .10 | -.10 |
| Better Overall Mood | .13 | -.12 | -.09 | -.02 | .15 | .08 | .08 | -.02 | .02 |
| Accomplishment | .12 | -.14 | -.19 | -.04 | -.04 | -.06 | .00 | .04 | -.05 |
| Depression | -.10 | .16 | .07 | -.04 | -.04 | .11 | .04 | -.01 | .04 |
| Anxiety | .29** | .23* | .28** | -.22** | -.06 | .09 | .08 | .11 | .11 |
| Stress | -.17 | .16 | .13 | .01 | .11 | .04 | .10 | -.07 | .01 |
| Physical Functioning | -.00 | .03 | .00 | -.04 | .08 | .17 | -.14 | -.04 | -.25* |
| Physical Role Limitations | -.05 | .17 | -.00 | -.02 | -.01 | -.02 | -.16 | -.06 | -.25* |
| Bodily Pain | .07 | -.02 | -.03 | .01 | -.01 | .05 | -.27** | -.09 | -.34** |
| General Health | .27** | .01 | .01 | .09 | .09 | -.14 | -.23* | .06 | -.36** |
| Vitality | .18 | -.12 | .10 | .04 | -.05 | -.04 | -.17 | .04 | -.16 |
| Social Functioning | .18 | -.10 | .06 | .08 | .03 | .06 | -.10 | .07 | -.08 |
| Emotional Role Limitations | .05 | -.09 | -.04 | -.05 | -.03 | -.06 | -.13 | -.02 | -.07 |
| Mental Health | .19* | .22* | -.07 | .03 | .07 | -.07 | -.06 | .10 | -.03 |

Supplementary Table 2. Correlation table for physical activity and all variables. * Significance p = 0.05; ** significance p = 0.01.

|  | Pre-pregnancy physical activity | Pregnancy physical activity | Post-pregnancy physical activity |
| --- | --- | --- | --- |
| Age |  |  |  |
| Relationship Status |  |  |  |
| Occupation |  |  |  |
| Education |  |  |  |
| Parity |  |  |  |
| Child Age |  |  |  |
| Pre-pregnancy BMI |  |  |  |
| Gestational Weight Gain |  |  |  |
| Post-pregnancy BMI |  |  |  |
| Pre-pregnancy Physical Activity | 1 |  |  |
| Pregnancy Physical Activity | .75** | 1 |  |
| Post-pregnancy Physical Activity | .55** | .57** | 1 |
| Vegetables (Fresh) | -.17 | -.19* | -.17 |
| Vegetables (Frozen) | -.12 | -.17 | -.19* |
| Fruit (Fresh) | -.07 | -.17 | -.05 |
| Fruit (Frozen) | -.20* | .23* | -.11 |
| Grains | .10 | .03 | .08 |
| Meat | .21* | .16 | .21* |
| Legumes | -.20* | .21* | -.16 |
| Dairy | .04 | -.03 | -.08 |
| Snacks | .03 | .07 | .09 |
| Hours of Sleep (Total) | -.01 | .14 | .12 |
| Hours of Sleep (Uninterrupted) | .01 | .09 | .15 |
| Number of Sleep Interruptions | .04 | -.09 | -.06 |
| Hours Awake at Night | .06 | -.07 | -.09 |
| Socialising with friends | -.08 | -.05 | -.13 |
| Getting Out of the House | .14 | .19* | .17 |
| Meet New People | .14 | .06 | -.01 |
| Praise | .11 | .06 | .07 |
| Weight Control | .08 | .07 | .14 |
| Increase Fitness | .25** | .27** | .23* |
| Reduce the risk of Type 2 Diabetes | .13 | .14 | .11 |
| More Energy | .21* | .24* | .09 |
| Feel Better Physically | .16 | .16 | .20* |
| Reduce Stress | .14 | -.01 | .08 |
| Better Overall Mood | .13 | -.02 | .03 |
| Accomplishment | .08 | .09 | .01 |
| Depression | .03 | -.13 | .08 |
| Anxiety | -.04 | -.26** | -.15 |
| Stress | .05 | -.11 | .13 |
| Physical Functioning | .09 | .13 | .23* |
| Physical Role Limitations | .07 | .06 | .03 |
| Bodily Pain | -.02 | .09 | .19* |
| General Health | .14 | .25** | .22* |
| Vitality | .03 | .19* | .15 |
| Social Functioning | -.00 | .12 | -.06 |
| Emotional Role Limitations | -.03 | .13 | -.03 |
| Mental Health | .07 | .17 | .03 |

Supplementary Table 3. Correlation table for dietary intake and all variables. * Significance p = 0.05; ** significance p = 0.01.

|  | Vegetables (Fresh) | Vegetables (Frozen) | Fruit (Fresh) | Fruit (Frozen) | Grains | Meat | Legumes | Dairy | Snacks |
| --- | --- | --- | --- | --- | --- | --- | --- | --- | --- |
| Age |  |  |  |  |  |  |  |  |  |
| Relationship Status |  |  |  |  |  |  |  |  |  |
| Occupation |  |  |  |  |  |  |  |  |  |
| Education |  |  |  |  |  |  |  |  |  |
| Parity |  |  |  |  |  |  |  |  |  |
| Child Age |  |  |  |  |  |  |  |  |  |
| Pre-pregnancy BMI |  |  |  |  |  |  |  |  |  |
| Gestational Weight Gain |  |  |  |  |  |  |  |  |  |
| Post-pregnancy BMI |  |  |  |  |  |  |  |  |  |
| Pre-pregnancy Physical Activity |  |  |  |  |  |  |  |  |  |
| Pregnancy Physical Activity |  |  |  |  |  |  |  |  |  |
| Post-pregnancy Physical Activity |  |  |  |  |  |  |  |  |  |
| Vegetables (Fresh) | 1 |  |  |  |  |  |  |  |  |
| Vegetables (Frozen) | -.04 | 1 |  |  |  |  |  |  |  |
| Fruit (Fresh) | .38** | .01 | 1 |  |  |  |  |  |  |
| Fruit (Frozen) | .22* | .23* | .20* | 1 |  |  |  |  |  |
| Grains | .12 | -.04 | .04 | .09 | 1 |  |  |  |  |
| Meat | .07 | -.03 | -.03 | -.01 | .11 | 1 |  |  |  |
| Legumes | .39** | -.12* | .34** | .35** | .02 | .06 | 1 |  |  |
| Dairy | -.08 | .04 | .01 | .11 | .11 | .14 | -.10 | 1 |  |
| Snacks | -.09 | .15 | -.06 | -.00 | .32** | .14 | -.05 | .16 | 1 |
| Hours of Sleep (Total) | -.12 | .07 | -.22* | -.21* | -.03 | .09 | -.14 | -.01 | .13 |
| Hours of Sleep (Uninterrupted) | -.04 | -.04 | -.08 | -.21* | .03 | .07 | -.15 | -.11 | .19* |
| Number of Sleep Interruptions | .07 | .05 | .17 | .03 | .07 | -.12 | .14 | .11 | -.06 |
| Hours Awake at Night | -.01 | -.09 | .12 | -.04 | -.01 | -.15 | .09 | -.12 | -.21* |
| Socialising with friends | -.18 | .04 | -.22* | -.01 | .01 | -.06 | -.16 | -.03 | .04 |
| Getting Out of the House | -.08 | -.09 | -.12 | -.04 | .09 | .01 | -.12 | .03 | .17 |
| Meet New People | -.02 | -.02 | -.04 | .07 | .22* | -.12 | -.14 | .01 | .16 |
| Praise | .11 | -.17 | -.11 | -.11 | .11 | .22* | -.11 | -.06 | .09 |
| Weight Control | .13 | -.14 | .22* | .10 | .17 | -.01 | .08 | -.04 | .15 |
| Increase Fitness | -.03 | -.06 | -.07 | .03 | .19* | -.00 | -.04 | -.01 | .19* |
| Reduce the risk of Type 2 Diabetes | -.05 | -.08 | -.02 | .04 | .09 | -.18 | -.11 | -.08 | .06 |
| More Energy | .07 | -.08 | -.02 | .02 | .20* | -.01 | -.07 | -.01 | .21* |
| Feel Better Physically | .06 | -.04 | .07 | .06 | .10 | .12 | -.01 | -.12 | .11 |
| Reduce Stress | .01 | .17 | -.01 | -.01 | .11 | -.00 | -.04 | -.01 | .22* |
| Better Overall Mood | -.04 | .07 | .01 | .11 | .10 | .09 | .04 | -.03 | .17 |
| Accomplishment | -.14 | .08 | -.05 | .00 | -.00 | -.07 | -.01 | -.01 | .11 |
| Depression | .26** | .02 | .30** | .04 | -.01 | -.01 | .15 | -.07 | -.00 |
| Anxiety | .32** | .01 | .45** | .03 | .12 | -.03 | .21* | -.03 | .03 |
| Stress | .29** | .06 | .34** | .10 | .02 | .11 | .17 | -.03 | .00 |
| Physical Functioning | -.10 | .10 | -.19* | .00 | .09 | -.09 | -.09 | -.12 | .27** |
| Physical Role Limitations | -.08 | .02 | -.18 | -.03 | -.02 | -.04 | .01 | -.10 | .19* |
| Bodily Pain | -.23* | .05 | -.21* | -.00 | .02 | -.03 | -.15 | .02 | .22* |
| General Health | -.32** | .54 | -.37** | -.14 | .08 | .07 | -.30** | -.02 | .22* |
| Vitality | -.21* | -.07 | -.33** | -.24* | .17 | -.05 | -.15 | -.02 | .17 |
| Social Functioning | -.26** | .09 | -.49** | -.78 | .08 | .08 | -.21* | .02 | .07 |
| Emotional Role Limitations | .23* | .01 | -.24** | -.09 | .17 | -.11 | -.12 | .16 | .12 |
| Mental Health | -.26** | -.12 | -.32** | -.16 | .12 | .03 | -.27** | .05 | .15 |

Supplementary Table 4. Correlation table for sleep and all variables. * Significance p = 0.05; ** significance p = 0.01.

|  | Hours of Sleep (Total) | Hours of Sleep (Uninterrupted) | Number of Sleep Interruptions | Hours Awake at Night |
| --- | --- | --- | --- | --- |
| Age |  |  |  |  |
| Relationship Status |  |  |  |  |
| Occupation |  |  |  |  |
| Education |  |  |  |  |
| Parity |  |  |  |  |
| Child Age |  |  |  |  |
| Pre-pregnancy BMI |  |  |  |  |
| Gestational Weight Gain |  |  |  |  |
| Post-pregnancy BMI |  |  |  |  |
| Pre-pregnancy Physical Activity |  |  |  |  |
| Pregnancy Physical Activity |  |  |  |  |
| Post-pregnancy Physical Activity |  |  |  |  |
| Vegetables (Fresh) |  |  |  |  |
| Vegetables (Frozen) |  |  |  |  |
| Fruit (Fresh) |  |  |  |  |
| Fruit (Frozen) |  |  |  |  |
| Grains |  |  |  |  |
| Meat |  |  |  |  |
| Legumes |  |  |  |  |
| Dairy |  |  |  |  |
| Snacks |  |  |  |  |
| Hours of Sleep (Total) | 1 |  |  |  |
| Hours of Sleep (Uninterrupted) | .44** | 1 |  |  |
| Number of Sleep Interruptions | -.40** | -.44** | 1 |  |
| Hours Awake at Night | -.50** | -.51** | .51** | 1 |
| Socialising with friends | .23* | .19* | -.19 | -.04 |
| Getting Out of the House | .12 | .10 | -.04 | .01 |
| Meet New People | -.05 | -.05 | -.01 | .08 |
| Praise | .19* | .13 | -.08 | -.07 |
| Weight Control | -.03 | .06 | .11 | -.06 |
| Increase Fitness | .11 | .15 | -.06 | -.18 |
| Reduce the risk of Type 2 Diabetes | -.07 | .13 | -.00 | -.09 |
| More Energy | .07 | .21* | -.17 | -.20* |
| Feel Better Physically | .28** | .27** | -.27** | -.30** |
| Reduce Stress | -.02 | -.02 | -.04 | -.09 |
| Better Overall Mood | -.01 | -.01 | -.16 | -.12 |
| Accomplishment | .23* | .09 | -.13 | -.11 |
| Depression | -.23* | -.10 | .14 | .22* |
| Anxiety | -.28** | -.04 | .14 | .19 |
| Stress | -.23* | -.12 | .13 | .17 |
| Physical Functioning | .16 | .14 | -.09 | -.18 |
| Physical Role Limitations | .14 | .06 | .09 | -.05 |
| Bodily Pain | .19 | .07 | -.13 | -.27** |
| General Health | .17 | .11 | -.02 | -.17 |
| Vitality | .43** | .32** | -.22* | -.44** |
| Social Functioning | .32** | .14 | -.17 | -.24* |
| Emotional Role Limitations | .24* | .12 | -.08 | -.23* |
| Mental Health | .27** | .19* | -.18 | -.27** |

Supplementary Table 5. Correlation table for health values and all variables. * Significance p = 0.05; ** significance p = 0.01.

|  | Socialising with friends | Getting out of the House | Meet New People | Praise | Weight Control | Increase Fitness | Reduce the risk of Type 2 Diabetes | More Energy | Feel Better Physically | Reduce Stress | Better Overall Mood | Accomplishment |
| --- | --- | --- | --- | --- | --- | --- | --- | --- | --- | --- | --- | --- |
| Age |  |  |  |  |  |  |  |  |  |  |  |  |
| Relationship Status |  |  |  |  |  |  |  |  |  |  |  |  |
| Occupation |  |  |  |  |  |  |  |  |  |  |  |  |
| Education |  |  |  |  |  |  |  |  |  |  |  |  |
| Parity |  |  |  |  |  |  |  |  |  |  |  |  |
| Child Age |  |  |  |  |  |  |  |  |  |  |  |  |
| Pre-pregnancy BMI |  |  |  |  |  |  |  |  |  |  |  |  |
| Gestational Weight Gain |  |  |  |  |  |  |  |  |  |  |  |  |
| Post-pregnancy BMI |  |  |  |  |  |  |  |  |  |  |  |  |
| Pre-pregnancy Physical Activity |  |  |  |  |  |  |  |  |  |  |  |  |
| Pregnancy Physical Activity |  |  |  |  |  |  |  |  |  |  |  |  |
| Post-pregnancy Physical Activity |  |  |  |  |  |  |  |  |  |  |  |  |
| Vegetables (Fresh) |  |  |  |  |  |  |  |  |  |  |  |  |
| Vegetables (Frozen) |  |  |  |  |  |  |  |  |  |  |  |  |
| Fruit (Fresh) |  |  |  |  |  |  |  |  |  |  |  |  |
| Fruit (Frozen) |  |  |  |  |  |  |  |  |  |  |  |  |
| Grains |  |  |  |  |  |  |  |  |  |  |  |  |
| Meat |  |  |  |  |  |  |  |  |  |  |  |  |
| Legumes |  |  |  |  |  |  |  |  |  |  |  |  |
| Dairy |  |  |  |  |  |  |  |  |  |  |  |  |
| Snacks |  |  |  |  |  |  |  |  |  |  |  |  |
| Hours of Sleep (Total) |  |  |  |  |  |  |  |  |  |  |  |  |
| Hours of Sleep (Uninterrupted) |  |  |  |  |  |  |  |  |  |  |  |  |
| Number of Sleep Interruptions |  |  |  |  |  |  |  |  |  |  |  |  |
| Hours Awake at Night |  |  |  |  |  |  |  |  |  |  |  |  |
| Socialising with friends | 1 |  |  |  |  |  |  |  |  |  |  |  |
| Getting Out of the House | .39** | 1 |  |  |  |  |  |  |  |  |  |  |
| Meet New People | .36** | .26** | 1 |  |  |  |  |  |  |  |  |  |
| Praise | .08 | .14 | .28** | 1 |  |  |  |  |  |  |  |  |
| Weight Control | .01 | .09 | .01 | .45** | 1 |  |  |  |  |  |  |  |
| Increase Fitness | .02 | .31** | .03 | .40** | .64** | 1 |  |  |  |  |  |  |
| Reduce the risk of Type 2 Diabetes | -.03 | .00 | .12 | .20* | .30** | .33** | 1 |  |  |  |  |  |
| More Energy | .08 | .10 | .07 | .21* | .51** | .54** | .43** | 1 |  |  |  |  |
| Feel Better Physically | .15 | .21* | .06 | .19* | .46** | .60** | .20* | .69** | 1 |  |  |  |
| Reduce Stress | .14 | .14 | .04 | .09 | .36** | .34** | .27** | .43** | .36** | 1 |  |  |
| Better Overall Mood | .21* | .15 | .08 | .11 | .39** | .34** | .30** | .47** | .50** | .75** | 1 |  |
| Accomplishment | .23* | .26** | .13 | .29** | .30** | .38** | .28** | .42** | .54** | .48** | .56** | 1 |
| Depression | -.33** | -.19 | -.06 | -.08 | .00 | -.23* | -.07 | -.10 | -.11 | .07 | .05 | -.18 |
| Anxiety | -.34** | -.34** | -.09 | .00 | .12 | -.10 | -.03 | .06 | -.04 | .09 | -.01 | -.15 |
| Stress | -.29** | -.18 | -.160 | -.15 | .09 | -.08 | -.07 | .08 | .03 | .19* | .11 | -.15 |
| Physical Functioning | .03 | .13 | -.02 | .02 | .02 | .06 | .09 | .10 | .10 | .15 | .15 | .18 |
| Physical Role Limitations | .12 | .23* | .09 | -.02 | -.07 | .00 | .06 | -.10 | -.11 | -.04 | -.01 | .04 |
| Bodily Pain | .20* | .11 | .04 | -.06 | -.05 | -.01 | .08 | -.05 | -.03 | -.07 | -.02 | .08 |
| General Health | .20* | .15 | .05 | -.06 | -.02 | .30** | .09 | .06 | .06 | .14 | .03 | .14 |
| Vitality | .26** | .17 | .14 | .22* | .10 | .29** | .07 | .04 | .20* | .04 | -.03 | -.19 |
| Social Functioning | .33** | .08 | .12 | .09 | -.12 | .04 | .09 | .08 | .05 | -.07 | -.08 | .04 |
| Emotional Role Limitations | .08 | -.01 | .12 | .12 | -.07 | .10 | .12 | -.03 | -.06 | -.14 | -.18 | -.07 |
| Mental Health | .33** | .22* | .17 | .23* | .06 | .22* | .03 | .07 | .13 | -.04 | -.04 | .17 |

Supplementary Table 6. Correlation table for mental health and all variables. * Significance p = 0.05; ** significance p = 0.01.

|  | Depression | Anxiety | Stress |
| --- | --- | --- | --- |
| Age |  |  |  |
| Relationship Status |  |  |  |
| Occupation |  |  |  |
| Education |  |  |  |
| Parity |  |  |  |
| Child Age |  |  |  |
| Pre-pregnancy BMI |  |  |  |
| Gestational Weight Gain |  |  |  |
| Post-pregnancy BMI |  |  |  |
| Pre-pregnancy Physical Activity |  |  |  |
| Pregnancy Physical Activity |  |  |  |
| Post-pregnancy Physical Activity |  |  |  |
| Vegetables (Fresh) |  |  |  |
| Vegetables (Frozen) |  |  |  |
| Fruit (Fresh) |  |  |  |
| Fruit (Frozen) |  |  |  |
| Grains |  |  |  |
| Meat |  |  |  |
| Legumes |  |  |  |
| Dairy |  |  |  |
| Snacks |  |  |  |
| Hours of Sleep (Total) |  |  |  |
| Hours of Sleep (Uninterrupted) |  |  |  |
| Number of Sleep Interruptions |  |  |  |
| Hours Awake at Night |  |  |  |
| Socialising with friends |  |  |  |
| Getting Out of the House |  |  |  |
| Meet New People |  |  |  |
| Praise |  |  |  |
| Weight Control |  |  |  |
| Increase Fitness |  |  |  |
| Reduce the risk of Type 2 Diabetes |  |  |  |
| More Energy |  |  |  |
| Feel Better Physically |  |  |  |
| Reduce Stress |  |  |  |
| Better Overall Mood |  |  |  |
| Accomplishment |  |  |  |
| Depression | 1 |  |  |
| Anxiety | .60** | 1 |  |
| Stress | .73** | .72** | 1 |
| Physical Functioning | -.13 | -.28** | -.16 |
| Physical Role Limitations | -.16 | -.34** | -.19* |
| Bodily Pain | -.27** | -.39** | -.30** |
| General Health | -.45** | -.41** | -.37** |
| Vitality | -.52** | -.35** | -.48** |
| Social Functioning | -.51** | -.45** | -.52** |
| Emotional Role Limitations | -.55** | -.42** | -.56** |
| Mental Health | -.73** | -.60** | -.77** |

Supplementary Table 7. Correlation table for wellness and all variables. * Significance p = 0.05; ** significance p = 0.01.

|  | Physical Functioning | Physical Role Limitations | Bodily Pain | General Health | Vitality | Social Functioning | Emotional Role Limitations | Mental Health |
| --- | --- | --- | --- | --- | --- | --- | --- | --- |
| Age |  |  |  |  |  |  |  |  |
| Relationship Status |  |  |  |  |  |  |  |  |
| Occupation |  |  |  |  |  |  |  |  |
| Education |  |  |  |  |  |  |  |  |
| Parity |  |  |  |  |  |  |  |  |
| Child Age |  |  |  |  |  |  |  |  |
| Pre-pregnancy BMI |  |  |  |  |  |  |  |  |
| Gestational Weight Gain |  |  |  |  |  |  |  |  |
| Post-pregnancy BMI |  |  |  |  |  |  |  |  |
| Pre-pregnancy Physical Activity |  |  |  |  |  |  |  |  |
| Pregnancy Physical Activity |  |  |  |  |  |  |  |  |
| Post-pregnancy Physical Activity |  |  |  |  |  |  |  |  |
| Vegetables (Fresh) |  |  |  |  |  |  |  |  |
| Vegetables (Frozen) |  |  |  |  |  |  |  |  |
| Fruit (Fresh) |  |  |  |  |  |  |  |  |
| Fruit (Frozen) |  |  |  |  |  |  |  |  |
| Grains |  |  |  |  |  |  |  |  |
| Meat |  |  |  |  |  |  |  |  |
| Legumes |  |  |  |  |  |  |  |  |
| Dairy |  |  |  |  |  |  |  |  |
| Snacks |  |  |  |  |  |  |  |  |
| Hours of Sleep (Total) |  |  |  |  |  |  |  |  |
| Hours of Sleep (Uninterrupted) |  |  |  |  |  |  |  |  |
| Number of Sleep Interruptions |  |  |  |  |  |  |  |  |
| Hours Awake at Night |  |  |  |  |  |  |  |  |
| Socialising with friends |  |  |  |  |  |  |  |  |
| Getting Out of the House |  |  |  |  |  |  |  |  |
| Meet New People |  |  |  |  |  |  |  |  |
| Praise |  |  |  |  |  |  |  |  |
| Weight Control |  |  |  |  |  |  |  |  |
| Increase Fitness |  |  |  |  |  |  |  |  |
| Reduce the risk of Type 2 Diabetes |  |  |  |  |  |  |  |  |
| More Energy |  |  |  |  |  |  |  |  |
| Feel Better Physically |  |  |  |  |  |  |  |  |
| Reduce Stress |  |  |  |  |  |  |  |  |
| Better Overall Mood |  |  |  |  |  |  |  |  |
| Accomplishment |  |  |  |  |  |  |  |  |
| Depression |  |  |  |  |  |  |  |  |
| Anxiety |  |  |  |  |  |  |  |  |
| Stress |  |  |  |  |  |  |  |  |
| Physical Functioning | 1 |  |  |  |  |  |  |  |
| Physical Role Limitations | .59** | 1 |  |  |  |  |  |  |
| Bodily Pain | .62** | .45** | 1 |  |  |  |  |  |
| General Health | .32** | .24* | .47** | 1 |  |  |  |  |
| Vitality | .23* | .17 | .42** | .51** | 1 |  |  |  |
| Social Functioning | .11 | .18 | .35** | .39** | .56** | 1 |  |  |
| Emotional Role Limitations | .10 | .21* | .25** | .32** | .54** | .54** | 1 |  |
| Mental Health | .13 | .19 | .32** | .39** | .64** | .61** | .62** | 1 |
